# Microbial Characteristics and Genomic Analysis of an ST11 Carbapenem-Resistant *Klebsiella pneumoniae* Strain Carrying *bla*_*KPC*−2_ Conjugative Drug-Resistant Plasmid

**DOI:** 10.3389/fpubh.2021.809753

**Published:** 2022-01-27

**Authors:** Lingyi Zeng, Jisheng Zhang, Kewang Hu, Jie Li, Jianmin Wang, Chengru Yang, Wan Huang, Lining Yin, Xiaoli Zhang

**Affiliations:** ^1^Department of Microbiology, Yongchuan Hospital of Chongqing Medical University, Chongqing, China; ^2^Department of Molecular Biology, Jiaxing Maternal and Child Health Hospital, Jiaxing, China; ^3^Department of Microbiology, Affiliated Hangzhou Xixi Hospital, Zhengjiang University School of Medicine, Hangzhou, China; ^4^Department of Microbiology, The First Affiliated Hospital of Jiamusi University, Jiamusi, China

**Keywords:** carbapenem-resistant *Klebsiella pneumoniae*, ST11, whole-genome sequencing, nanopore, KPC-2

## Abstract

**Background:**

The sequence type 11 (ST11) carbapenem-resistant *Klebsiella pneumoniae* (CRKP) carrying *bla*_*KPC*−2_ has been widespread all over the world, and it has been reported frequently in China. The *bla*_*KPC*−2_ located on the mobile genetic element brings tremendous pressure to control the spread and outbreak of resistant bacteria. Whole-genome sequencing (WGS) technology can comprehensively and in-depth display the molecular characteristics of drug-resistant bacteria, providing a basis for evaluating the genetic diversity within the CRKP genome.

**Methods:**

The ST11 CRKP in this study was collected in the intensive care unit of a major teaching hospital. PCR and Sanger sequencing confirmed the existence of *bla*_*KPC*−2_. The AST-GN card and the microbroth dilution test were used for antimicrobial susceptibility testing. The transferability of plasmid was verified by a conjugation test. The whole genome is sequenced using the Illumina HiSeq short-read and Oxford Nanopore long-read sequencing technology.

**Results:**

The studied strain was named CRKP63, which is a multi-drug resistance bacteria, which carries *bla*_*KPC*−2_ and *bla*_*SHV*−182_. Its genome consists of a circular chromosome of 5,374,207 bp and an IncFII plasmid named pKPC-063001 of 359,625 bp. In the drug-resistant plasmid pKPC-063001, the key carbapenem resistance gene *bla*_*KPC*−2_ was located in the genetic context with insertion sequence IS*Kpn27* upstream and IS*Kpn6* downstream and bracketed by IS*26*. The three copies of the IS*26*–IS*Kpn27*–*bla*_*KPC*−2_–IS*Kpn6*–IS*26* unit were present in tandem. *bla*_*KPC*−2_ can be transferred horizontally between other species by conjugation, the complete type IV secretion system (T4SS) structure helps to improve the adaptability of bacteria to the external environment, strengthen the existence of drug-resistant bacteria, and accelerate the spread of drug resistance.

**Conclusion:**

High-throughput sequencing has discovered the different surrounding environments of *bla*_*KPC*−2_, which provides a new idea for further revealing the transmission and inheritance of *bla*_*KPC*−2_ at the molecular level. In order to control the further spread and prevalence of drug-resistant bacteria, we should pay close attention to the changes in the genetic environment of *bla*_*KPC*−2_ and further study the transcription and expression of T4SS.

## Background

Carbapenem-resistant *Enterobacteriaceae*, especially *Klebsiella pneumoniae*, have emerged as important causes of morbidity and mortality among hospital-acquired and long-term care-associated infections (1). As of now, carbapenem-resistant *K. pneumoniae* (CRKP) strains have spread worldwide and posed a severe threat to public health. *K. pneumoniae* carbapenemases (*KPC*)*-2*, the most common variant of *KPC* enzymes, is a dominant factor mediating carbapenems resistance in CRKP (2, 3). The most predominant isolates of *KPC*-producing *K. pneumoniae* (*KPC-kpn*) belong to the clonal group 258 (CG258), two representative types of this CG: the ST258 and ST11 strains, have been identified worldwide – ST258 is mostly prevalent in America and Europe, while ST11 is the highly dominant clone in Asia (especially in China) (4).

The *bla*_*KPC*−2_ is a typical plasmid-mediated drug resistance gene and mainly carried on plasmids of different incompatibility (Inc) groups, such as IncFII, FIA, I2, A/C, N, X, P, and L/M (4). The *bla*_*KPC*−2_ on the plasmid can spread the resistance through different methods, such as gene duplication, transposon elements, or plasmid transfer. The horizontal transmission of drug-resistant plasmids can accelerate the spread of multidrug resistance genes and mediate the production of multidrug-resistant bacteria (MDR). An in-depth understanding of the plasmid structure and its genome characteristics will help to control and prevent the emergence and outbreak of drug-resistant bacteria.

The rapid development of whole-genome sequencing (WGS) technology has gradually matured its application in the field of clinical microbiology (5, 6). WGS has the characteristics of large data information and high resolution, and it plays an important value in the research and detection of MDR. In this study, a whole-genome sequence of a CRKP, which is ST11 type and carrying *bla*_*KPC*−2_ in Chongqing, China, was performed and further explored its microbiological and genomic characteristics.

## Materials and Methods

### Bacterial Collection

According to previous research, a total of 51 non-duplicated CRKP samples isolated from the ICU of Yongchuan Affiliated Hospital of Chongqing Medical University (a major teaching hospital in Chongqing, China) were collected from July 2018 to July 2020. Homology analysis based on the result of pulsed-field gel electrophoresis showed that 62.7% of the isolates belonged to the same cluster, indicating that there was a clonal transmission of ST11 carrying *bla*_*KPC*−2_ CRKP in the ICU of this hospital. In order to further study the molecular characteristics of CRKP in this ward, we selected one of the strains from the clone group, which carries multiple drug resistance genes and has the ability to conjugate—CRKP63—and conduct in-depth research on it. This *bla*_*KPC*−2_-positive isolate was collected after identification (VITEK-2 automated microbiology analyzer, bioMérieux, France) and routine antimicrobial susceptibility testing by the Microbiology Laboratory in April 2020. The strain was identified as *bla*_*KPC*−2_ producing carbapenem-resistant *K. pneumoniae* by PCR detection and drug sensitivity (carbapenems) review. The isolate was stored at −80°C for further study.

### Antimicrobial Susceptibility Testing

The VITEK-2 Compact automatic microbiological analyzer Antimicrobial Susceptibility Testing-Gram-Negtive (AST-GN) card (bioMérieux, France) was used for routine antimicrobial susceptibility testing. Minimum inhibitory concentration (MIC) is defined as the lowest compound concentration (μg/ml) required to stop bacterial growth was determined by using the microbroth dilution method. Imipenem (IPM), meropenem (MEM), amikacin (AMK), levofloxacin (LEV), tigecycline (TIG), polymyxin B (PLB), and ceftazidime-avibactam (CAZ-AVI) were used to determine the MIC by the microbroth dilution method. ATCC 25922, ATCC 700603, and BAA-1705 were used as quality control strains. Three parallel assays were performed for each sample. The IPM, MEM, AMK, LEV, PLB, and CAZ-AVI results were interpreted based on the Clinical and Laboratory Standards Institute (CLSI) criteria (7), whereas the TIG results were interpreted based on the European Committee on Antimicrobial Susceptibility Testing (EUCAST) (8) breakpoint recommendations.

### Conjugation Experiment

The conjugation experiment was carried out using a membrane bonding experiment as previously described (9). Both the donor (CRKP) and the recipient strains (*E. coli EC600*) were mixed in Luria-Bertani broth at a ratio of 1:3, and the mixtures were placed on a membrane and incubated for 24 h at 35°C. Transconjugants were selected on Mueller-Hinton agar II (MHA) plates supplemented with rifampicin (600 μg/ml) and MEM (1 μg/ml). Colonies that grew on the selective medium were identified by the VITEK-2 Compact system and *16S rRNA* sequence. A strain that harbored carbapenemase and exhibited higher MICs of resistance to carbapenems than *EC600* was defined as the transconjugants and the presence of resistance determinants was confirmed by PCR.

### WGS and Data Analysis

Genomic DNA was isolated using the MagAttract HMW DNA Kit (Qiagen, Hilden, Germany) and submitted to next-generation high-throughput sequencing (NGS) on a HiSeq 2000™ platform (Illumina Inc., San Diego, CA, USA) with 2 × 100-bp paired-end reads and to long-read high-throughput sequencing (LRS) on a MinION platform (Oxford Nanopore Technologies, Oxford, UK). The long reads generated by MinION were assembled using Canu v. 1.6 (10) and polished with the short reads generated by HiSeq using Pilon v1.22 (11) to obtain the whole genome and complete plasmid sequences. The chromosome and plasmid sequences were annotated using the prokaryotic gene prediction tool Prokka (12). The plasmid incompatibility type was searched using the online tool PlasmidFinder (https://cge.cbs.dtu.dk//services/PlasmidFinder/) (13). Antibiotic resistance genes were identified using both the Comprehensive Antibiotic Resistance Database (CARD) (14) and ResFinder database (https://cge.cbs.dtu.dk/services/ResFinder/) (15). Meanwhile, virulence-associated genes were identified using VirulenceFinder (https://cge.cbs.dtu.dk/services/VirulenceFinder/) (16). Transposon and insertion sequence (IS) elements were scanned using the ISfinder database (https://www-is.biotoul.fr/) (17). Comparative plasmid illustration was implemented by BRIG (http://brig.sourceforge.net) (18) or Easyfig tools (https://github.com/mjsull/Easyfig) (19). BLAST (https://blast.ncbi.nlm.nih.gov/Blast.cgi) (20) was used for comparative analysis through the coverages and identities.

### Nucleotide Sequence Accession Numbers

The complete sequences were submitted to GeneBank under accession numbers.

## Result

### Clinical Character

**Figure 1:**
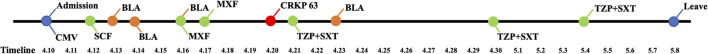
The main process of the patient during hospitalization. CMV, mechanical ventilation; BLA, bronchoalveolar lavage; SCF, cefoperazone/sulbactam; MXF, moxifloxacin; TZP, piperacillin tazobactam; SXT, sulfamethoxazole.

The *bla*_*KPC*−2_-positive CRKP, CRKP63, collected in this study was derived from a 90-year-old female patient in the ICU ward. Concurrently, this strain also carried *bla*_*SHV*−182_. The patient was admitted to the hospital due to acute exacerbation of chronic obstructive pulmonary disease on April 10, 2020, on the day of admission, the patient underwent mechanical ventilation. The patient underwent bronchoalveolar lavage 4 times during the hospitalization, and 10 days after admission, *K. pneumoniae* was detected in the bronchoalveolar lavage fluid, which was identified as a carbapenemase-producing CRKP. During the patient's hospitalization, infectious symptoms were repeatedly realized and anti-infective treatment continued. Twenty-eight days after admission, the condition of the patient did not improve, and finally, the patient and the patient's family gave up treatment and was discharged voluntarily. The main process of the patient during hospitalization is shown in Figure 1.

### Result of Antimicrobial Susceptibility

Carbapenem-resistant *Klebsiella pneumoniae*63 shows resistance to more than three antibiotics and can be defined as MDR (21). The MIC value of IPM and MEM was as high as 256 μg/ml. However, it was sensitive to tobramycin (TOB), sulfamethoxazole (SXT), TIG, and CAZ-AVI. The specific information of various antibiotics is shown in Table 1.

**Table 1:** Susceptibility results of various antibiotics (μg/ml).

**Isolate**	**Resistance genes**	**Susceptibility results of various antibiotics (μg/ml)**										
**CRKP isolate**												
CRKP 63	*KPC-2 SHV-182*	AMK	AMP	SAM	ATM	CZO	FEP	CTT	CAZ	CRO	CXM	CIP
		≤1/2	>16	>16	>32	>32	>32	>32	>32	>32	6	>2
		GEN	IPM	LVX	NIT	TZP	TOB	SXT	MEM	TIG	PB	CAZ-AVI
		≤1	256	32	256	>64	≤1	≤1	256	1	2	4, 4
***E. col*** **transconjugant strain**												
CRKP*J63*	*KPC-2*	AMK	AMP	SAM	ATM	CZO	FEP	CTT	CAZ	CRO	CXM	CIP
		≤1/2	>16	>16	>32	16	8	32	16	>32	4	≤1/4
		GEN	IPM	LVX	NIT	TZP	TOB	SXT	MEM	TIG	PB	CAZ-AVI
		≤1	4	1	≤16	>64	≤1	≤1	4	1	≤1/2	1/2, 4

*AMK, amikacin; AMP, ampicillin; SAM, ampicillin/sulbactam; ATM, aztreonam; CZO, cefazolin; FEP, cefepime; CTT, cefotetan; CAZ, ceftazidime; CRO, ceftriaxone; CXM, cefuroxime; CIP, ciprofloxacin; GEN, gentamicin; IPM, imipenem; LVX, levofloxacin; NIT, nitrofurantoin; TZP, piperacillin/sulbactam; TOB, tobramycin; SXT, sulfamethoxazole; MEM, meropenem; TIG, tigecycline; PB, polymyxin B; CAZ-AVI, ceftazidime/avibactam*.

### Conjugation Experiment

After the conjugation experiment was successful, the transconjugant CRKP*J63* was obtained. Compared with the original donor, CRKP*J63* still carried *bla*_*KPC*−2_, but not carried *bla*_*SHV*−182_. Obviously, the MIC value of transconjugant for carbapenems (IMP and MEM) was significantly decreased. The variation on donor and transconjugant susceptibility profiles is shown in Table 1.

### Results of WGS

**Figure 2:**
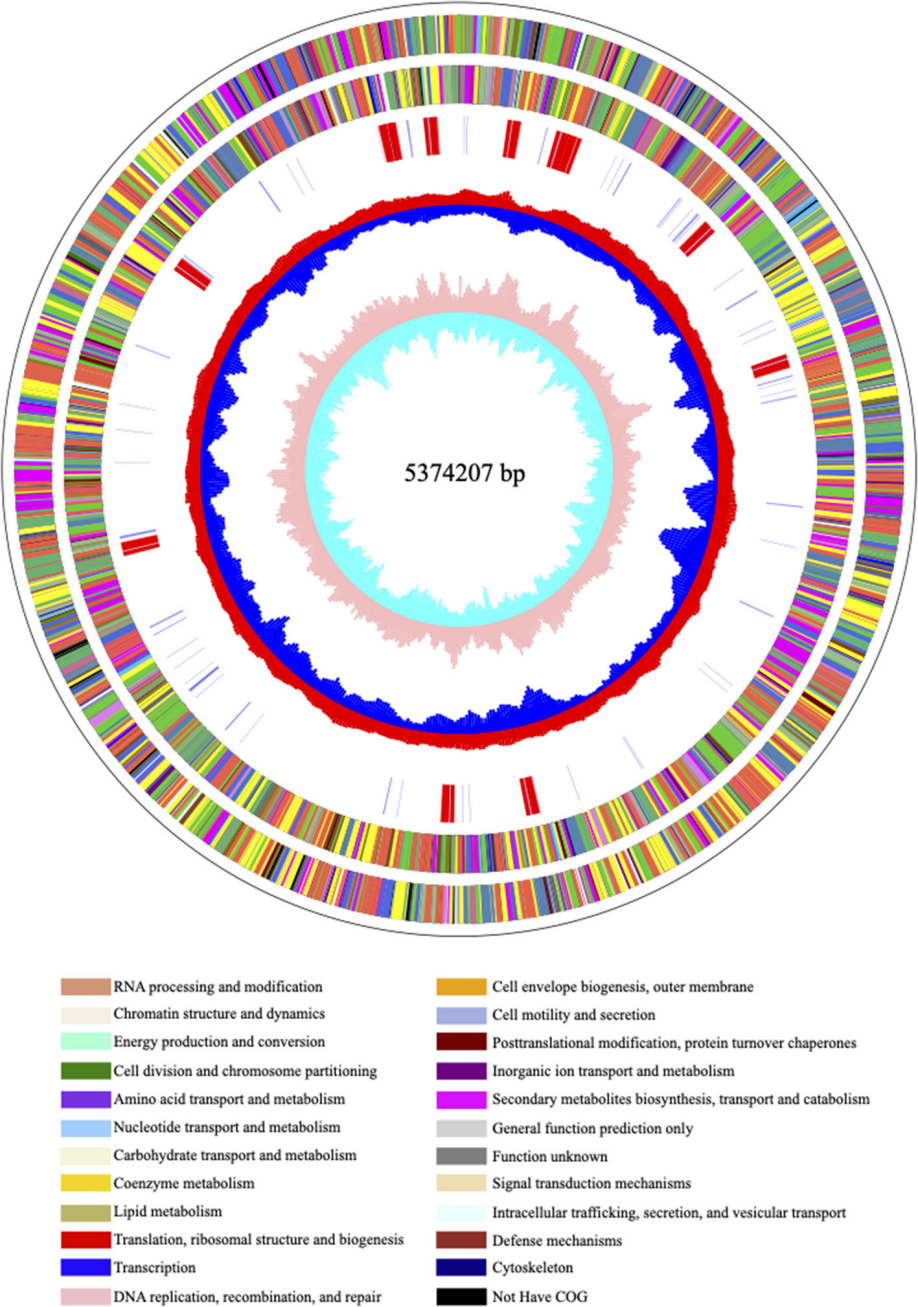
The visual circle map of the chromosome. From outside to inside, the first and second circles are CDS on the positive and negative strands, the third circle is rRNA and tRNA; the fourth circle is the GC content, and the outward red part indicates that the GC content of this region is higher than the average GC content of the whole genome. The higher the peak, the greater the difference from the average GC content, the inward blue part indicates that the GC content of the region is lower than the average GC content of the whole genome, the higher the peak, the greater the difference from the average GC content; the innermost circle (fifth) is the GC skew value, the specific algorithm is G – C/G + C, when the value is positive in the biological sense, the positive chain is more inclined to transcribe CDS, which is a negative value (22, 23).

**Figure 3:**
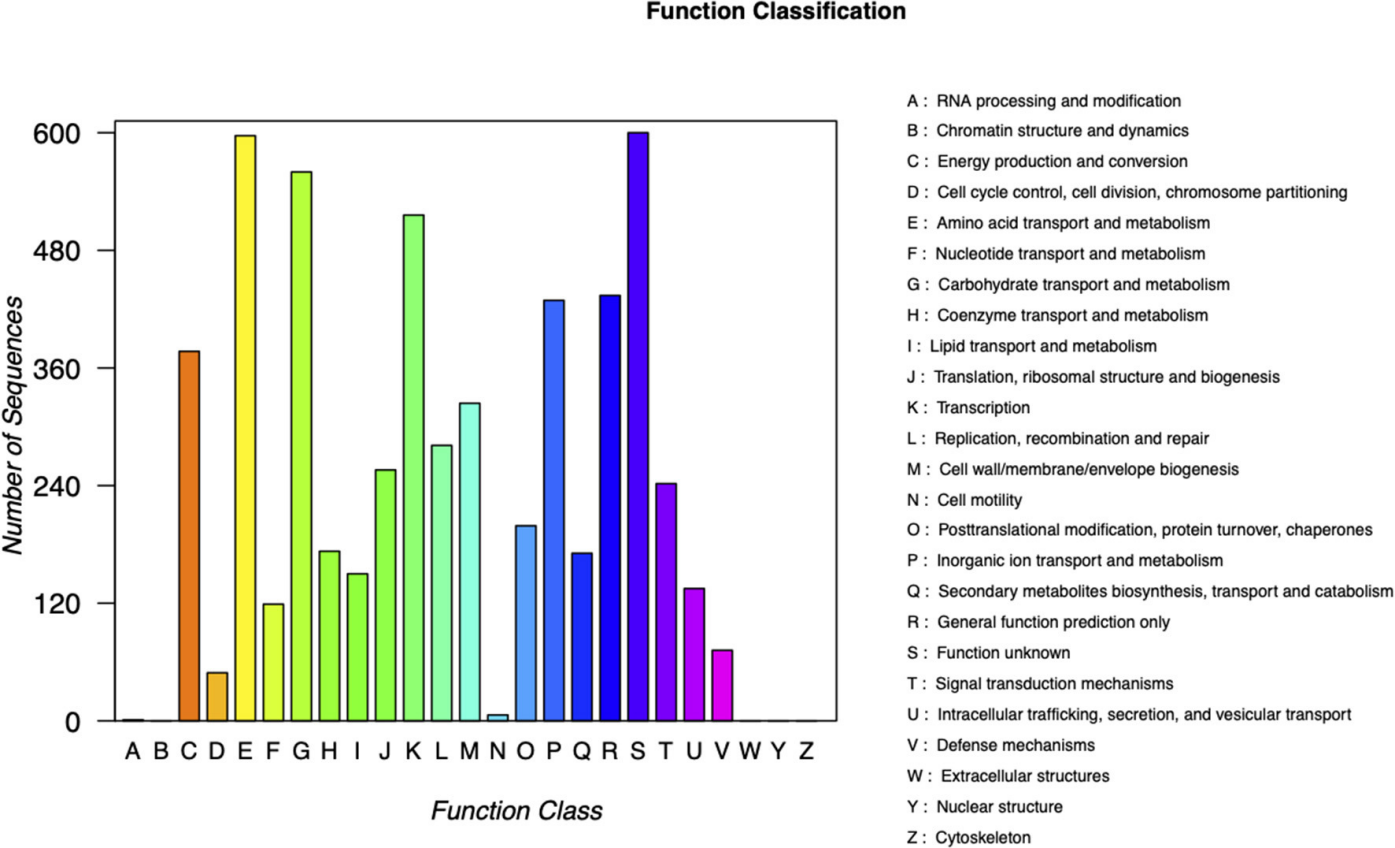
Classification of COG functions of chromosome in DNA libraries. COG, Cluster of Orthologous Groups.

Carbapenem-resistant *Klebsiella pneumoniae*63 carries two drug resistance genes at the same time, *bla*_*KPC*−2_ and *bla*_*SHV*−182_. The extended-spectrum β-lactamase (ESBL) gene *bla*_*SHV*−182_ is located on the chromosome (position in contig 1,037,173–1,038,033). Yet, the key carbapenem resistance gene *bla*_*KPC*−2_ is located on the drug resistance plasmid pKPC-063001, which is of type IncFII. Therefore, the whole genome of CRKP63 consists of a circular chromosome of 5,374,207 bp and a drug-resistant plasmid (named pKPC-063001) of 359,625 bp. For chromosome, the final draft genome showed a G + C content of 60.4%, with a total of 5,165 annotated protein-coding sequences (CDSs). The visual circle map is shown in Figure 2. The Cluster of Orthologous Groups [(COGs) (of proteins)] database was used to annotate its genome, and it was found that genes related to metabolism and genes related to genetic information processing accounted for a relatively large proportion. In addition, there are also functional proteins related to gene processing and material conversion. The class of protein function and its number are shown in Figure 3.

**Figure 4:**
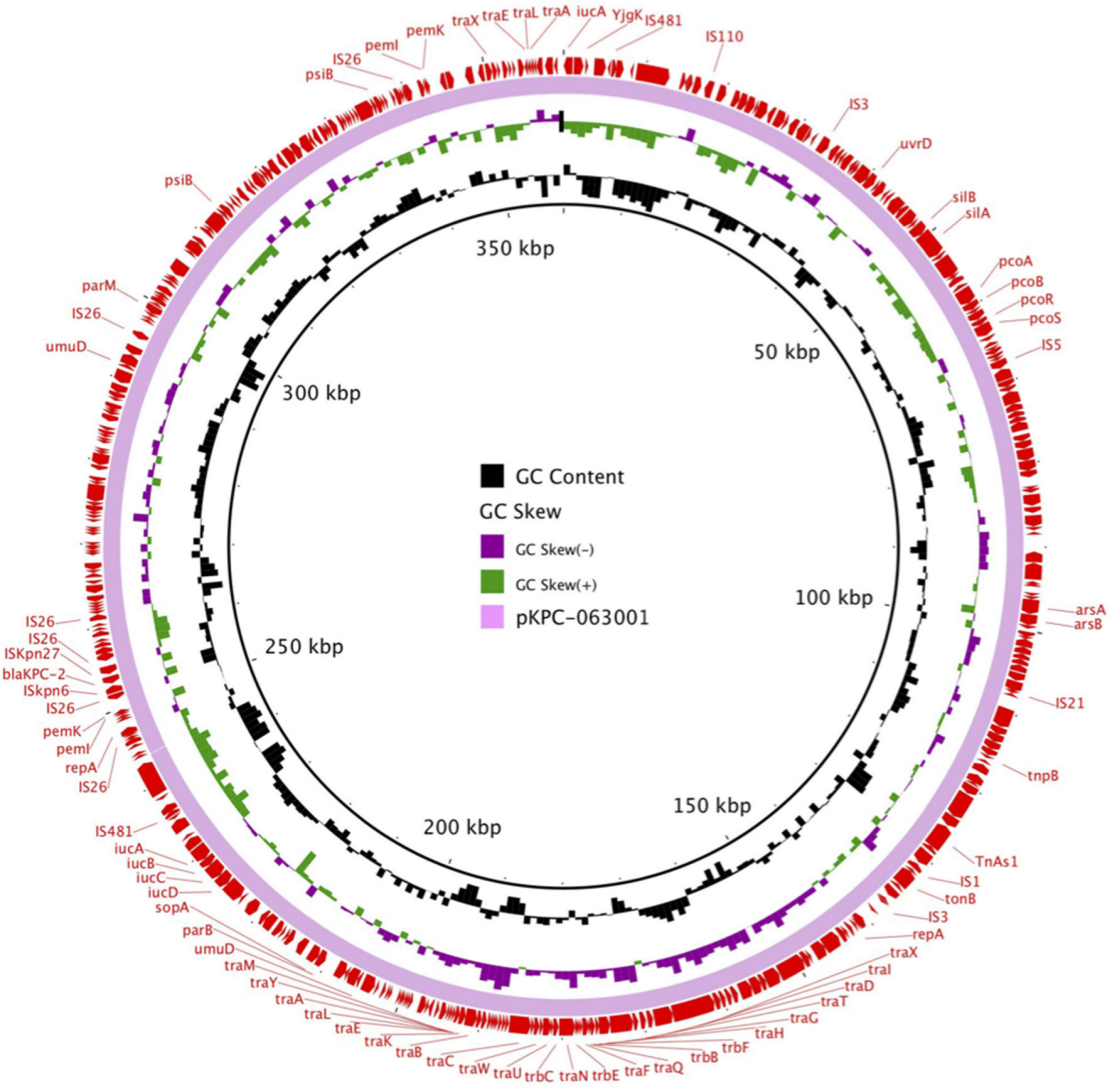
The visual circle map of plasmid pKPC-063001, where *bla*_*KPC*−2_ is located. GC content, GC skew+, and GC skew– are, respectively, indicated in black, green, and purple.

**Figure 5:**
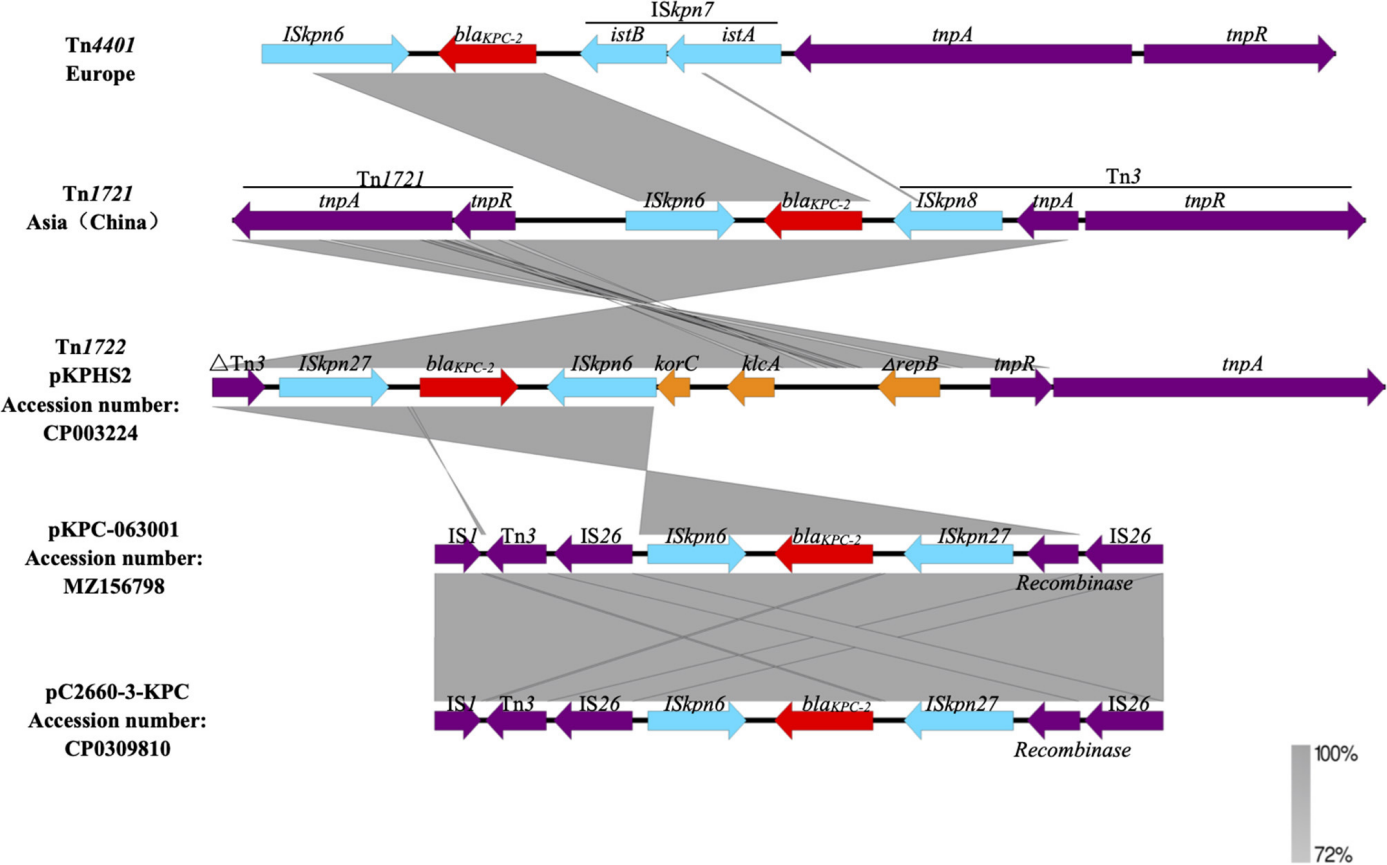
Comparison of the surrounding environment of *bla*_*KPC*−2_.

pKPC-063001 is a 359,625 bp circular molecule with an average G + C content of 58.19% and was predicted to encode a total of 409 CDSs. In addition to the *bla*_*KPC*−2_, it also contains virulence factors *iucA, iucB, iucC*, and *iucD*, plasmid replication protein (*repA*), plasmid stabilization protein (*parA*), and the type IV secretion system (T4SS) proteins *traA, traB, traD, traM*, and *traK* that mediate the conjugation and transfer of plasmids. The visual circle map is shown in Figure 4. After a detailed analysis of the surrounding structure of the key gene *bla*_*KPC*−2_, it was found that *bla*_*KPC*−2_ is located in a gene fragment with IS*26* repeat inserts at both ends. This gene fragment has IS*26* repeats at both ends, and IS*kpn27* and IS*kpn6* in the middle, and there is also a *Tn3-tnpR* structure between IS*kpn27* and IS*26* (Figure 5).

### Accession Numbers

The result obtained by sequencing in this study has been uploaded to the Genebank website (www.ncbi.nlm.nih.gov). The accession number of the chromosome is CP077763 (GenBank: CP077763.1), and the accession number of the plasmid is MZ156798 (GenBank: MZ156798.1).

## Discussion

The *bla*_*KPC*_ gene still plays a key role in high-level carbapenem resistance (24). One of the key factors contributing to the rapid and wide dissemination of *bla*_*KPC*−2_ is its location on a transposable element (25, 26). In Europe, *bla*_*KPC*−2_ is mainly located on the conserved Tn*3* family transposon Tn*4401* (25) and is considered the origin of the acquisition and dissemination of this marker (27). Tn*4401* is approximately 10 kb in size and delimited by two 39 bp imperfect inverted repeat sequences. It harbors two ISs flanking *bla*_*KPC*_, IS*Kpn6*, and IS*Kpn7*, in addition to transposase (*tnpA*) and resolvase (*tnpR*) genes. However, Tn*1721*-like transposons among ST11 *K. pneumoniae* are mainly responsible for the effective spread of the *bla*_*KPC*−2_ gene in China (28). There are three inverted repeats, *IRR, IRL1*, and *IRL2*, in the Tn*1721a* transposon. *tnpA* and *tnpR* are located between *IRR* and *IRL1*, while the 5′ end of *bla*_*KPC*−2_ has a complete Tn*3* located between *IRL1* and *IRL2*. When Tn*3*-tnp R is interrupted by IS*26*, Tn*3*-tnp A and *IRL2* are lost, and a new structure, such as Tn*1721*-*bla*_*KPC*−2_-ΔTn*3*-IS*26* (Tn*1721b*), is formed. The Tn*1722*-based unit transposons with the IS*Kpn27*-*bla*_*KPC*−2_-ΔIS*Kpn6* core structure (IS*Kpn27* is initially named in the ISfinder database) has been also reported in China, such as pKP048 (29), p628-KPC (30), and pHS102707.

In this study, the surrounding environment of the *bla*_*KPC*−2_ is different from the epidemic structure of domestic and international, we call it “composite transposon-based on IS*26*”. The original 1319bp IS*Kpn6* was truncated by IS*26* and became 881bp ΔIS*Kpn6*. IS*Kpn27* is quite different from other inserted sequences, and there is a “recombinase” gene between IS*Kpn27* and IS*26* repeats (Figure 5). Notably, different lengths of truncated IS*Kpn6* can be observed for these IS*26*-based transposons from different plasmids. This structure has also been reported by others (31), such as pC2660-3-KPC (32). This difference in transposons indicates that there is a certain variability and diversity surrounding the environment of the *bla*_*KPC*−2_ gene, which may also be one of the factors causing the rapid spread of *bla*_*KPC*−2_ strain and the different epidemic status in different regions. Loftie et al. (33) found that Tn*6231* can significantly improve the persistence of resistant plasmid pMS0506 and broaden the host range of plasmid, which shows that the evolutionary behavior of plasmid (such as transposon) can affect the spread of resistance gene and improve the selection of plasmid host. Mutations in the genetic environment help to improve the durability of antibiotic resistance, so that the host adapts to changes in the external environment and then affects the spread of bacterial resistance. Therefore, the *bla*_*KPC*−2_ gene environment is constantly changing, and its horizontal transfer with transposable elements shows greater flexibility than plasmid transmission. In this way, the clinical harm will be greater.

As the key carbapenemase, *bla*_*KPC*−2_ can exist on many different types of plasmids. The *bla*_*KPC*_ found in this study is located on the IncFII type plasmid. The success of the conjugation experiment also confirmed the conjugability of the plasmid. It can be seen that the pKPC-063001 has a complete functional skeleton structure, i.e., plasmid replication structure, plasmid stabilization structure, and plasmid conjugation transfer structure. These structures provide a structural basis for the widespread of *bla*_*KPC*−2_. Among them, theT4SS plays an important role in conjugative transfer (34, 35). The classic T4SS system was originated from *Agrobacterium rhizogenes*, it was generally composed of 12 proteins (i.e., 11 *VirB* proteins and 1 *VirD* coupling protein (*VirD4*)) (36, 37), coupling protein–relaxosome contact could lead to DNA unwinding, generating a single strand of DNA that is then transferred to the recipient in a 5′ to 3′ direction (38), so that the genetic information is transferred. Then, T4SS was also found in Gram-negative bacteria (39). Among the two major phylogenetic groups of gram-negative bacteria T4SSs, type IVA (the conjugation systems of the IncF and IncP plasmids) is more common (40, 41). Lawley et al. (34) reported that T4SSs, also known as the mating pair formation (Mpf) apparatus, are central to the dissemination of numerous genetic determinants between bacteria, as highlighted by the spread of antibiotic resistance among pathogens. After studying the gene deletion of the T4SS regulatory region of the pCTX-M3 plasmid of the IncM group, Dmowski et al. found that (42) the knockout of the conjunction structural gene will result in no transfer of the resistance gene or low conjunction rate. However, when *orf35* and *orf36* were knocked out, the plasmid conjugation rate could be improved, this is because two genes are involved in suppressing the transcriptional regulation of the T4SS gene according to transcription analysis. This shows that the existence of T4SS undoubtedly provides strong support for the global popularity of *bla*_*KPC*−2_. The complete T4SS structure helps to improve the adaptability of bacteria to the external environment, thereby enhancing the existence of drug-resistant bacteria and accelerating the spread of drug-resistant bacteria. An in-depth study of the function and transcriptional expression of T4SS will help prevent the further spread of *bla*_*KPC*−2_.

In addition, it should be noted that *bla*_*SHV*−182_ located on the chromosome did not transfer with *bla*_*KPC*−2_, simultaneously, the results of conjugation showed that the MIC value of conjugants for carbapenems decreased, indicating that other causes of drug resistance, such as membrane protein, or efflux pump, for example, did not transfer with the plasmid. This also suggests that the resistance of CRKP63 is caused by a variety of factors, not simply caused by pKPC-063001.

Combined with previous research (43), we know that CRKP63 was collected in the ICU ward of a hospital in Chongqing, China. There had been an outbreak of ST11 CRKP carrying *bla*_*KPC*−2_ in this ward. This outbreak was closely related to the horizontal transfer of *bla*_*KPC*−2_. Similarly, over the past period, IncFII plasmids carrying *bla*_*KPC*−2_ often have been reported in *K. pneumoniae* in China, especially the ST11 (44), the prevalence and dissemination of InFII plasmid carrying *KPC-2* in China has become a fact. However, unlike the dominant Tn*1721* in China, the presence of “IS*26*-based composite transposon” structure represented by pKPC-063001 implies the variability and complexity of *bla*_*KPC*−2_. In the future, “IS*26*-based composite transposon” is likely to become the dominant clone group in China and even the world with amazing speed and adaptability, thence, continuous monitoring will be necessary to prevent further dissemination of pKPC-063001 type plasmid.

In conclusion, this study reported the microbial and genomic characteristics of an ST11 CRKP carrying *bla*_*KPC*−2_ in Chongqing, China. Through WGS, we found the different surrounding environments of *bla*_*KPC*−2_, which provides a new research idea for further revealing the transmission and inheritance of *bla*_*KPC*−2_ at the molecular level. The differences in the surrounding environment of *bla*_*KPC*−2_ create convenience for its dissemination and popularity, and the complete T4SS structure provides a solid guarantee for it. Therefore, in order to control the further spread and prevalence of drug-resistant bacteria, we should also pay close attention to the genetic environment of *bla*_*KPC*−2_, and further study the transcription and expression of T4SS.

## Data Availability Statement

The datasets presented in this study can be found in online repositories. The names of the repository/repositories and accession number(s) can be found below: https://www.ncbi.nlm.nih.gov/genbank/, CP077763 and https://www.ncbi.nlm.nih.gov/genbank/, MZ156798.

## Ethics Statement

The studies involving human participants were reviewed and approved by The Ethics Committee of Yongchuan Hospital of ChongQing Medical University. Written informed consent for participation was not required for this study in accordance with the national legislation and the institutional requirements.

## Author Contributions

XZ conceived and designed the study. LZ and JZ wrote this paper and contributed equally to this work. LZ, JW, CY, and WH performed the experiments. JL, KH, LY, and LZ analyzed the data. All authors contributed to the article and approved the submitted version.

## Funding

This work was supported by the General Projects of Chongqing Natural Science Foundation (cstc2020jcyj-msxm0067), the Yongchuan Natural Science Foundation (2021yc-jckx20053), and the Talent introduction project of Yongchuan Hospital of Chongqing Medical University (YJYJ202005 and YJYJ202004).

## Conflict of Interest

The authors declare that the research was conducted in the absence of any commercial or financial relationships that could be construed as a potential conflict of interest.

## Publisher's Note

All claims expressed in this article are solely those of the authors and do not necessarily represent those of their affiliated organizations, or those of the publisher, the editors and the reviewers. Any product that may be evaluated in this article, or claim that may be made by its manufacturer, is not guaranteed or endorsed by the publisher.

## References

[1] Munoz-Price LS Poirel L Bonomo RA Schwaber MJ Daikos GL Cormican M. Clinical epidemiology of the global expansion of *Klebsiella pneumoniae* carbapenemases. Lancet Infect Dis. (2013) 13:785–96. 10.1016/S1473-3099(13)70190-7 23969216 PMC4673667

[2] Nordmann P Cuzon G Naas T. The real threat of *Klebsiella pneumoniae* carbapenemase-producing bacteria. Lancet Infect Dis. (2009) 9:228–36. 10.1016/S1473-3099(09)70054-4 19324295

[3] Shi L Feng J Zhan Z Zhao Y Zhou H Mao H. Comparative analysis of bla *KPC-2-* and *rmtB*-carrying IncFII-family *pKPC-LK30*/*pHN7A8* hybrid plasmids from *Klebsiella pneumoniae* CG258 strains disseminated among multiple Chinese hospitals. Infect Drug Resist. (2018) 11:1783–93. 10.2147/IDR.S171953 30349335 PMC6188201

[4] Chen L Mathema B Chavda KD DeLeo FR Bonomo RA Kreiswirth BN. Carbapenemase-producing *Klebsiella pneumoniae*: molecular and genetic decoding. Trends Microbiol. (2014) 22:686–96. 10.1016/j.tim.2014.09.003 25304194 PMC4365952

[5] Fleischmann RD Adams MD White O Clayton RA Kirkness EF Kerlavage AR. Whole-genome random sequencing and assembly of Haemophilus influenzae Rd. Science. (1995) 269:496–512. 10.1126/science.7542800 7542800

[6] Ferrari C Corbella M Gaiarsa S Comandatore F Scaltriti E Bandi C. Multiple *Klebsiella pneumoniae KPC* clones contribute to an extended hospital outbreak. Front Microbiol. (2019) 10:2767. 10.3389/fmicb.2019.02767 31849904 PMC6896718

[7] CLSI. Performance Standards for Antimicrobial Suceptibility Testing. In: 30th ed. CLSI supplement M100. Wayne, PA: Clinical and Laboratory Standards Institute. (2020).

[8] The European Committee on Antimicrobial Susceptibility Testing. In: Breakpoint tables for interpretation of MICs and zone diameters, version 10.0. (2020).

[9] Gong X Zhang J Su S Fu Y Bao M Wang Y. Molecular characterization and epidemiology of carbapenem non-susceptible *Enterobacteriaceae* isolated from the Eastern region of Heilongjiang Province, China. BMC Infect Dis. (2018) 18:417. 10.1186/s12879-018-3294-3 30134844 PMC6106938

[10] Koren S Walenz BP Berlin K Miller JR Bergman NH Phillippy AM. Canu: scalable and accurate long-read assembly via adaptive k-mer weighting and repeat separation. Genome Res. (2017) 27:722–36. 10.1101/gr.215087.116 28298431 PMC5411767

[11] Walker BJ Abeel T Shea T Priest M Abouelliel A Sakthikumar S. Pilon: an integrated tool for comprehensive microbial variant detection and genome assembly improvement. PLoS ONE. (2014) 9:e112963. 10.1371/journal.pone.0112963 25409509 PMC4237348

[12] Seemann T. Prokka: rapid prokaryotic genome annotation. Bioinformatics. (2014) 30:2068–9. 10.1093/bioinformatics/btu153 24642063

[13] Carattoli A Zankari E Garcia-Fernandez A Voldby Larsen M Lund O Villa L. In silico detection and typing of plasmids using PlasmidFinder and plasmid multilocus sequence typing. Antimicrob Agents Chemother. (2014) 58:3895–903. 10.1128/AAC.02412-14 24777092 PMC4068535

[14] McArthur AG Waglechner N Nizam F Yan A Azad MA Baylay AJ. The comprehensive antibiotic resistance database. Antimicrob Agents Chemother. (2013) 57:3348–57. 10.1128/AAC.00419-13 23650175 PMC3697360

[15] Bortolaia V Kaas RS Ruppe E Roberts MC Schwarz S Cattoir V. ResFinder 4.0 for predictions of phenotypes from genotypes. J Antimicrob Chemother. (2020) 75:3491–500. 10.1093/jac/dkaa345 32780112 PMC7662176

[16] Malberg Tetzschner AM Johnson JR Johnston BD Lund O Scheutz Scheutz F: In silico genotyping of *Escherichia coli* isolates for extraintestinal virulence genes by use of whole-genome sequencing data. J Clin Microbiol. (2020) 58:e01269–20. 10.1128/JCM.01269-20 32669379 PMC7512150

[17] Siguier P Perochon J Lestrade L Mahillon J Chandler M: ISfinder: the reference centre for bacterial insertion sequences. Nucleic Acids Res. (2006) 34:D32–36. 10.1093/nar/gkj014 16381877 PMC1347377

[18] Alikhan NF Petty NK Ben Zakour NL Beatson SA. BLAST Ring Image Generator (BRIG): simple prokaryote genome comparisons. BMC Genomics. (2011) 12:402. 10.1186/1471-2164-12-402 21824423 PMC3163573

[19] Sullivan MJ Petty NK Beatson SA. Easyfig: a genome comparison visualizer. Bioinformatics. (2011) 27:1009–10. 10.1093/bioinformatics/btr039 21278367 PMC3065679

[20] Camacho C Coulouris G Avagyan V Ma N Papadopoulos J Bealer K. BLAST+: architecture and applications. BMC Bioinformatics. (2009) 10:421. 10.1186/1471-2105-10-421 20003500 PMC2803857

[21] Magiorakos AP Srinivasan A Carey RB Carmeli Y Falagas ME Giske CG. Multidrug-resistant, extensively drug-resistant and pandrug-resistant bacteria: an international expert proposal for interim standard definitions for acquired resistance. Clin Microbiol Infect. (2012) 18:268–81. 10.1111/j.1469-0691.2011.03570.x 21793988

[22] Lobry JR. Asymmetric substitution patterns in the two DNA strands of bacteria. Mol Biol Evol. (1996) 13:660–5. 10.1093/oxfordjournals.molbev.a025626 8676740

[23] Necsulea A Lobry JR. A new method for assessing the effect of replication on DNA base composition asymmetry. Mol Biol Evol. (2007) 24:2169–79. 10.1093/molbev/msm148 17646257

[24] Huang J Hu X Zhao Y Shi Y Ding H Xv J Ren J Wu R Zhao Z. Genetic Factors Associated with Enhanced *bla KPC* Expression in Tn*3*/Tn*4401* Chimeras. Antimicrob Agents Chemother. (2020) 64:e01836–19. 10.1128/AAC.01836-19 31844015 PMC7038255

[25] Naas T Cuzon G Villegas MV Lartigue MF Quinn JP Nordmann P. Genetic structures at the origin of acquisition of the beta-lactamase *bla KPC* gene. Antimicrob Agents Chemother. (2008) 52:1257–63. 10.1128/AAC.01451-07 18227185 PMC2292522

[26] Cuzon G Naas T Nordmann P. Functional characterization of Tn*4401*, a Tn*3*-based transposon involved in *blaKPC* gene mobilization. Antimicrob Agents Chemother. (2011) 55:5370–3. 10.1128/AAC.05202-11 21844325 PMC3195030

[27] Tzouvelekis LS Miriagou V Kotsakis SD Spyridopoulou K Athanasiou E Karagouni E. *KPC*-producing, multidrug-resistant *Klebsiella pneumoniae* sequence type 258 as a typical opportunistic pathogen. Antimicrob Agents Chemother. (2013) 57:5144–6. 10.1128/AAC.01052-13 23856769 PMC3811402

[28] Fu P Tang Y Li G Yu L Wang Y Jiang X. Pandemic spread of *blaKPC-2* among *Klebsiella pneumoniae* ST11 in China is associated with horizontal transfer mediated by IncFII-like plasmids. Int J Antimicrob Agents. (2019) 54:117–24. 10.1016/j.ijantimicag.2019.03.014 30885806

[29] Shen P Wei Z Jiang Y Du X Ji S Yu Y. Novel genetic environment of the carbapenem-hydrolyzing beta-lactamase *KPC-2* among *Enterobacteriaceae* in China. Antimicrob Agents Chemother. (2009) 53:4333–8. 10.1128/AAC.00260-09 19620332 PMC2764158

[30] Wang L Fang H Feng J Yin Z Xie X Zhu X. Complete sequences of *KPC-2*-encoding plasmid *p628-KPC* and *CTX-M-55*-encoding *p628-CTXM* coexisted in *Klebsiella pneumoniae*. Front Microbiol. (2015) 6:838. 10.3389/fmicb.2015.00838 26347725 PMC4541600

[31] Feng Y Liu L McNally A Zong Z. Coexistence of three *blaKPC-2* genes on an IncF/IncR plasmid in ST11 *Klebsiella pneumoniae*. J Glob Antimicrob Resist. (2019) 17:90–3. 10.1016/j.jgar.2018.11.017 30496820

[32] Gao H Liu Y Wang R Wang Q Jin L Wang H. The transferability and evolution of *NDM-1* and *KPC-2* co-producing *Klebsiella pneumoniae* from clinical settings. EBioMedicine. (2020) 51:102599. 10.1016/j.ebiom.2019.102599 31911273 PMC6948161

[33] Loftie-Eaton W Yano H Burleigh S Simmons RS Hughes JM Rogers LM. Evolutionary paths that expand plasmid host-range: implications for spread of antibiotic resistance. Mol Biol Evol. (2016) 33:885–97. 10.1093/molbev/msv339 26668183 PMC4840908

[34] Lawley TD Klimke WA Gubbins MJ Frost LS. F factor conjugation is a true type IV secretion system. FEMS Microbiol Lett. (2003) 224:1–15. 10.1016/S0378-1097(03)00430-0 12855161

[35] Cenens W Andrade MO Llontop E Alvarez-Martinez CE Sgro GG Farah CS. Bactericidal type IV secretion system homeostasis in Xanthomonas citri. PLoS Pathog. (2020) 16:e1008561. 10.1371/journal.ppat.1008561 32453788 PMC7286519

[36] Redzej A Ukleja M Connery S Trokter M Felisberto-Rodrigues C Cryar A. Structure of a VirD4 coupling protein bound to a VirB type IV secretion machinery. EMBO J. (2017) 36:3080–95. 10.15252/embj.201796629 28923826 PMC5916273

[37] Alvarez-Martinez CE Christie PJ. Biological diversity of prokaryotic type IV secretion systems. Microbiol Mol Biol Rev. (2009) 73:775–808. 10.1128/MMBR.00023-09 19946141 PMC2786583

[38] Ohki M Tomizawa J. Asymmetric transfer of DNA strands in bacterial conjugation. Cold Spring Harb Symp Quant Biol. (1968) 33:651–8. 10.1101/SQB.1968.033.01.074 4892002

[39] Costa TR Felisberto-Rodrigues C Meir A Prevost MS Redzej A Trokter M. Secretion systems in Gram-negative bacteria: structural and mechanistic insights. Nat Rev Microbiol. (2015) 13:343–59. 10.1038/nrmicro3456 25978706

[40] Christie PJ Atmakuri K Krishnamoorthy V Jakubowski S Cascales E. Biogenesis, architecture, and function of bacterial type IV secretion systems. Annu Rev Microbiol. (2005) 59:451–85. 10.1146/annurev.micro.58.030603.123630 16153176 PMC3872966

[41] Christie PJ. The mosaic type IV secretion systems. EcoSal Plus. (2016) 7. 10.1128/ecosalplus.ESP-0020-2015 27735785 PMC5119655

[42] Dmowski M Golebiewski M Kern-Zdanowicz I. Characteristics of the conjugative transfer system of the IncM plasmid pCTX-M3 and identification of its putative regulators. J Bacteriol. (2018) 200:e00234–18. 10.1128/JB.00234-18 29986941 PMC6112013

[43] Zeng L Yang C Zhang J Hu K Zou J Li J. An outbreak of carbapenem-resistant *Klebsiella pneumoniae* in an intensive care unit of a major teaching hospital in Chongqing, China. Front Cell Infect Microbiol. (2021) 11:656070. 10.3389/fcimb.2021.656070 34150672 PMC8208809

[44] Yu X Zhang W Zhao Z Ye C Zhou S Wu S. Molecular characterization of carbapenem-resistant *Klebsiella pneumoniae* isolates with focus on antimicrobial resistance. BMC Genomics. (2019) 20:822. 10.1186/s12864-019-6225-9 31699025 PMC6839148

